# Female Infertility and Risk for Later-Life Cardiovascular Disease: Lessons from a Mouse Model of Human Cardiovascular Disease

**DOI:** 10.1007/s43032-025-02026-y

**Published:** 2026-01-16

**Authors:** Ayaka Tanaka, Hitomi Nakamura, Namhyo Kim, Hajime Nakaoka, Makoto Nishida, Keiichi Kumasawa, Yasushi Sakata, Shizuya Yamashita, Tadashi Kimura

**Affiliations:** 1 Present Address: Department of Obstetrics and Gynaecology, Osaka University Graduate School of Medicine, 2-2 Yamadaoka, Suita, Osaka Japan; 2 Department of Cardiovascular Medicine, Osaka University Graduate School of Medicine, 2-2 Yamadaoka, Suita, Osaka Japan

**Keywords:** Cardiovascular disease (CVD), Female infertility, Uterine receptivity, Implantation failure, Apolipoprotein E, SR-BI (scavenger receptor class B, Type I)

## Abstract

The potential link between female infertility during reproductive age and an increased risk of cardiovascular disease (CVD) later in life remains controversial. Female fertility is a complex process involving multiple steps, and disruptions at any stage can result in infertility. If only a specific subset of fertility dysfunctions—often challenging to diagnose—is associated with future CVD risk, this relationship may be masked by the heterogeneity of infertility causes. Identifying infertility factors that predispose women to CVD requires careful selection of study populations. To address this, we investigated fertility status in scavenger receptor class B, type I-deficient mice with hypomorphic apolipoprotein E mice, which represent the dominant pathologic foundation for human CVD. These female mice were healthy and active when fed a normal chow diet at reproductive age, but generated no offspring. The dysfunctional female fertility processes identified included degeneration of oocytes during the maturation processes prior to ovulation and impairment of implantation via dysfunctional decidualisation. The administration of probucol, a hypocholesterolaemic agent, significantly decreased the plasma total cholesterol level, as well as the size of high-density lipoprotein-like particles, and these alterations restored female fertility. These findings suggest that infertility caused by degeneration of oocytes during the maturation processes and defective uterine receptivity may contribute to CVD risk later in life. Women with unexplained infertility and recurrent implantation failure may represent an important cohort for future CVD risk assessment.

**Supplementary Information:**

The online version contains supplementary material available at 10.1007/s43032-025-02026-y.

## Introduction

Cardiovascular disease (CVD) is an important public health problem. Several studies have previously reported that parity is associated with CVD risk in later life, even without any history of complications during pregnancy [1–3]. Surprisingly, future CVD risk in nulliparous and primiparous women is higher compared to women with a history of two prior pregnancies [4, 5]. The Dallas Heart Study supports these reports, as the lowest frequency of calcification of the coronary artery and hypertrophic aortic wall was observed in women with a history of two or three prior pregnancies. Conversely, women with a history of more than four prior pregnancies, as well as nulliparous and primiparous women, showed a higher frequency of calcification of the coronary artery and hypertrophic aortic wall [6]. However, it is important to be aware that not all nulliparous women have a history of infertility. Among nulliparous women who eventually experienced childbirth, the risk of CVD in women who experienced more than 5 years of subfertility, but not 1–2 or 3–4 years, is significantly higher compared with women who conceived within 1 year [7]. On the other hand, although the failure to ovulate is one of the most common overall causes of infertility in women, several reports have shown an unfavourable future CVD risk in women diagnosed with premature ovarian insufficiency (POI) [8–10] or polycystic ovary syndrome (PCOS) [11, 12], which affects 70% of women with anovulation [13, 14]. Women with a history of infertility are highly heterogenous as female fertility processes comprise multiple steps, the disruption of which at any step results in female infertility. We hypothesised that only a subset of the dysfunctional female fertility processes, which may be difficult to diagnose, would be relevant to future CVD risk. If so, we propose that this population could be masked by the majority of the infertile population in whom infertility was caused by other factors. The most common causes of infertility amongst couples are ovulatory dysfunction, male factor infertility, and tubal factor infertility [13]. However, approximately 15–25% of infertile cases are unexplained, which includes dysfunction of uterine receptivity [13, 15]. To ascertain whether some forms of female infertility may act as a predisposition for CVD in later-life, it is necessary to correctly select the appropriate population. However, how can we choose the appropriate population? The assessment of fertility status in a mouse model, representing human CVD, would help to find a candidate population for further cohort studies to assess whether certain causes of female infertility predispose patients to CVD in later-life.

The dominant pathologic foundation for human CVD is atherosclerosis and, with regards to coronary heart disease (CHD), coronary atherosclerosis and resulting lumen stenosis, even total occlusions. Even though many CHD mouse models have been described, few represent the dominant pathologic foundation for human CVD [16]. Apolipoprotein E (apoE) mediates the uptake of remnant lipoproteins in the liver by binding to the low-density lipoprotein (LDL) receptor [17, 18]. As murine models for atherosclerosis, LDL receptor-[19] and apoE-deficient mice [20–22] both exhibit atherosclerotic lesions in the aorta, but do not usually develop myocardial infarction. The high-density lipoprotein (HDL) receptor, scavenger receptor class B, type I (SR-BI), is known as an anti-atherogenic reverse cholesterol transporter as it selectively removes cholesterol esters from HDL [23], SR-BI-deficient mice on a low-fat diet show hypercholesterolemia, but no spontaneous atherosclerosis [24, 25]. In the reverse cholesterol transport pathway, apoE plays an auxiliary role in the cellular uptake of HDL [26]. SR-BI and apoE double knockout (dKO) mice develop coronary lesions, multiple myocardial infarctions, and cardiac dysfunction [27, 28]. Although in humans, CVD events generally occur sometime in later-life due to the long-term effects of lifestyle, this mouse model showed very strong atherogenicity during the early life stages, and all the mice died by reproductive age (8-weeks of age; 50% mortality at 6-weeks of age), even when fed a normal diet. However, instead of being completely deficient in apoE, hypomorphic apoE with SR-BI deficient (SR-BI KO/*ApoeR61*^h/h^) mice showed diet-induced hypercholesterolemia, coronary atherosclerosis, and CHD, but were healthy and active when fed a normal chow diet [29]. This mouse model has been used as a diet-induced human CVD model [29–31]. In this human CVD model, the male mice were fertile, but the female mice generated no offspring [29]. This mouse model could represent women with a history of infertility and a high risk of CVD in later-life. In this study, we aimed to investigate the dysfunctional fertility process associated with CVD and assess the pathophysiology of female infertility in human CVD model mice.

## Materials and Methods

### Animals

All animal experimentation procedures were conducted with approval by the Animal Experiments Committee of Osaka University (Permit number 26–033-005 and 24–001–018) and the Genetic Modification Experiments Safety Committee at Osaka University (Permit number 3333). Specific pathogen-free C57BL/6J mice were purchased from a commercial breeder (SLC, Shizuoka, Japan) for use in this study. SR-BI KO/*ApoeR61*^h/h^ mice (mixed C57BL/6 × 129 backgrounds) were kindly provided by Professor Monty Krieger (Department of Biology, Massachusetts Institute of Technology, USA) [29, 30]. The genotypes were determined by polymerase chain reaction, as previously reported [24, 32]. At 5 weeks of age, SR-BI KO/*ApoeR61*^h/h^ female mice were randomised (1:1) to be fed a normal chow diet containing 0.5% probucol (Sinlestal®, Alfresa Pharma Corporation, Osaka, Japan; probucol group) or a normal chow diet (placebo group) as a control. The animals were maintained under controlled conditions (temperature, 23 ± 1.5 °C; relative humidity, 45 ± 15%), with a 12-h light/dark cycle (lights were turned off at 8:00 pm and turned on at 8:00 am). At 8–10 weeks of age, the virgin female mice were examined using vaginal smears to determine their sexual phases, as described previously [33]. At di-oestrus, blood samples and ovarian tissues were collected from the 10-week-old mice after fasting for 6 h. Female mice in pro-oestrus and oestrus were mated with male wild-type mice. We minimised the number of animals used by performing a sample size calculation beforehand. Refinement was done by using proven techniques, performed by trained personnel. Each mouse was given an identification number to be assessed in a blinded-to-group allocation during the experiment and analysis.

### Measurement of Blood Lipid Profile and Serum Progesterone Level

Plasma total cholesterol and lipoprotein levels in each group were measured at Skylight Biotech Inc. (Akita, Japan) as previously described [34]. The serum progesterone levels on day 4.5 p.c. were measured using a mouse progesterone enzyme-linked immunosorbent assay kit (Alpco, NH, US) according to the manufacturer’s protocol.

### Follicular Analysis

The follicular analysis was performed as described in our previous report [35]. Briefly, the formalin-fixed ovarian tissues were used for histological analysis. The 4-μm paraffin-embedded tissue sections were stained with haematoxylin–eosin stain. The follicular classification was assessed as reported previously [36]. To avoid duplicate counting, only those follicles that included a visible oocyte nucleus were counted. The numbers of each type of follicle were quantified by taking a 1 mm^2^ area from anywhere in the 4-μm wide section, selected from every 5th section in the ovarian samples. The follicular density was calculated using the following formula:

Follicular density (/mm^3^) = numbers of each type of follicle counted/1 × 0.004 × 5 × N, where N is the number of sections examined per ovary.

### Oocyte Retrieval

Female SR-BI KO/*ApoeR61*^h/h^ mice were superovulated at 7–8 weeks of age with 5 IU intraperitoneal (i.p.) injections of gonadotropin from pregnant mare's serum (PMSG, Sigma-Aldrich, MO, USA) and human chorionic gonadotropin (hCG, Sigma-Aldrich, MO, USA) in a 48-h time period. Oocytes were collected from the oviduct 12.5 to 14 h after hCG injection. The numbers of metaphase II oocytes were assessed in each group (*n* = 7).

### Analysis of Implantation

Blastocysts were flushed out of the uterus on day 4.0 p.c. from the probucol and placebo groups, and their numbers were assessed. On day 4.5 p.c., pregnant animals received an intravenous injection of 0.5% Evans Blue 15 min prior to sacrifice to visualise implantation sites [37].

### Experimental Decidualisation

To induce experimental decidualisation, pseudo-pregnant female mice mated with vasectomised wild-type male mice received an intraluminal infusion of 10 μl of sesame oil in one uterine horn (the contralateral horn served as a control) on day 3.5 p.c. Seventy two hours after treatment, fold increases in uterine weights of oil-infused horns over that of non-infused horns were used as an index of decidualisation, as described by Rider et al. [38].

### Immunohistochemistry

On day 4.5 p.c., the uterine tissues were removed and fixed in formalin. The uteri from wild-type female mice were used as a control. The 5-μm paraffin-embedded tissue sections were immunostained using monoclonal rabbit anti-mouse phosphorylated signal transducer and activator of transcription-3 (p-Stat3) antibody (Tyr705) (#9145; Cell Signaling Technology, Tokyo, Japan) at 1:400 dilution, polyclonal rabbit anti-mouse leukaemia inhibitory factor (LIF) antibody (OABF00432; Aviva Systems biology, CA, US) at 1:400 dilution, polyclonal goat anti-mouse leptin receptor antibody (AF497; R&D Systems, MN, US) at 1:200 dilution, and polyclonal rabbit anti-mouse cyclooxygenase (COX) −2 antibody (#160,126; Cayman Chemical, MI, US) at 1:500 dilution according to the manufacturers’ instructions. For each immunohistochemistry assay, rabbit (X0936; Dako, Agilent, Tokyo, Japan) or goat immunoglobulin G (#02–6202; Zymed, Thermo Fisher Scientific, Tokyo, Japan) at the same concentration as the primary antibody were used as a negative control. Biotinylated goat anti-rabbit immunoglobulins (Histofine #426,011, Nichirei Bioscience, Tokyo, Japan) or rabbit anti-goat immunoglobulins (E0466; Dako) were used for secondary antibody incubation, followed by detection with 3, 3’-diaminobenzidine tetrahydrochloride. Nuclear staining was performed with haematoxylin for light microscopy analysis using Intelligent Microscope (BX63, Olympus Scientific Solutions, Tokyo Japan).

### Statistical Analysis

The data were statistically analysed using the JMP® Pro 13 for Windows software (SAS Institute Inc.) and SigmaPlot® software 10.01 (Systat Software, Inc, San Jose, CA, USA). Data were analysed using Student’s *t*-test or the Mann–Whitney U test with the Shapiro–Wilk normality test, and differences with a *P*-value less than 0.05 were considered significant.

## Results

### Deletion of the SR-BI Gene with Hypomorphic apoE (*ApoeR61*^h/h^) Led to Female Infertility, but Probucol Administration Rescued the Impairment of Fertility

**Fig. 1:**
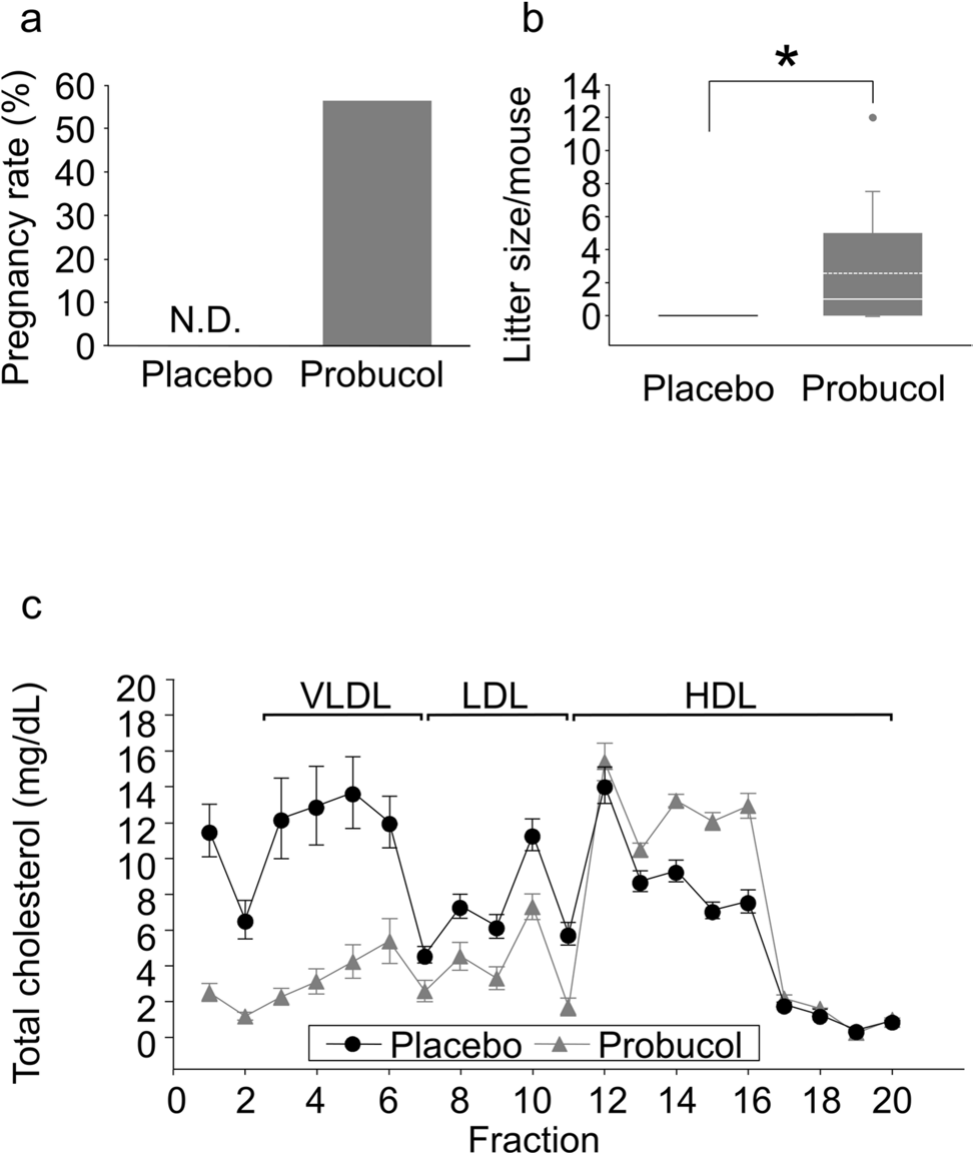
Fertility in SR-BI KO/*ApoeR61*^*h/h*^ female mice and effects of probucol administration. Virgin SR-BI KO/*ApoeR61*^*h/h*^ female mice with a normal chow diet only (placebo group, black bar and box, *n* = 19) or containing 0.5% probucol (probucol group; grey bar and box, *n* = 23) were housed with wild-type males for 2 months. The percentage pregnancy rate (**a**) and the number of foetuses per mouse (**b**) were assessed in each group. The horizontal line within the box shows the median, and the dotted line within the box shows the mean value. • Represents outliers. Data were evaluated using Shapiro–Wilk normality test and Mann–Whitney U test (*P < 0.05). Plasma samples were collected from SR-BI KO/*ApoeR61*^h/h^ female mice fed a normal chow diet with or without probucol at 10 weeks of age after fasting for 6 h, and the profiles of plasma lipoprotein total cholesterol (**c**) were assessed (*n* = 5 each). Abbreviations: VLDL, very low-density lipoprotein; LDL, low-density lipoprotein; HDL, high-density lipoprotein

None of the human CVD model (SR-BI KO/*ApoeR61*^h/h^) female mice mated to wild-type males became pregnant (0/19) even though intercourse was observed (Fig. 1a). Dysregulated lipid metabolism is known to be one of the important contributing factors to CVD. The administration of probucol, which is used for the prevention of atherosclerotic CVD, significantly reduced plasma levels of total cholesterol, very low-density lipoprotein cholesterol (VLDL-C) and LDL-C as expected, and significantly increased plasma HDL-C levels compared to those in the placebo group (Supplementary Fig. 1). Moreover, the size of HDL-like particles in the probucol group was slightly smaller than those in the placebo group (Fig. 1c). This alteration allowed 56.5% (13/23) of the human CVD model mice to become pregnant (Fig. 1a), resulting in viable offspring (2.6 ± 3.4, mean ± standard deviation [SD], *P* < 0.005, Mann–Whitney U test, Fig. 1b).

### What is the Dysfunctional Female Fertility Process in the Human CVD Model Mouse, and is there a Dysfunctional Fertility Process during Oocyte Development?

Oocytes grow inside the follicles in the ovary. During oocyte development, the size and cellular morphology of the follicles change until ovulation [39]. In mice, mating occurs within a very limited period, which is oestrus, and mating induces ovulation [39]. To assess whether (i) low frequency of oestrus and/or (ii) disruption of follicular development (early stages of oocyte maturation before ovulation) results in female infertility, which was recovered by probucol administration, the oestrous cycle and histology of follicular development were assessed in the human CVD mouse model.

**Fig. 2:**
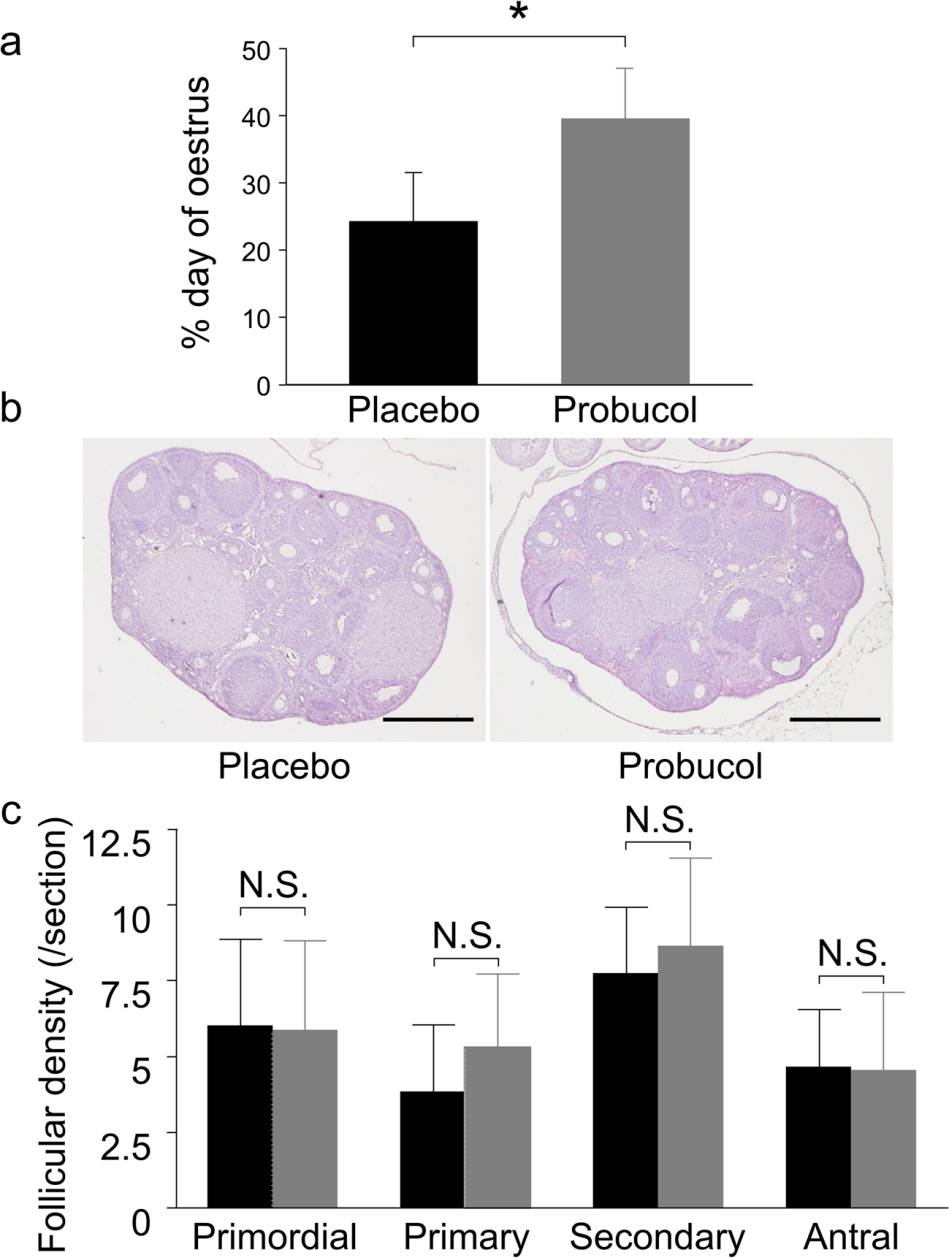
Analysis of the oestrus cycle and follicular population in the ovary. The percentage of consecutive days of oestrus (**a**) was analysed in SR-BI KO/*ApoeR61*^*h/h*^ mice with a normal chow diet (placebo group; black bar, *n* = 5) and a normal chow diet containing 0.5% probucol (probucol group; grey bar, *n* = 6). Data were evaluated using Shapiro–Wilk normality test and Student’s *t*-test (*P < 0.05). Haematoxylin and eosin staining of ovaries from the placebo (left) and probucol (right) groups at di-oestrus (**b**). Follicular density at each follicular stage in the placebo (black bar, *n* = 5) and probucol (grey bar, *n* = 5) groups at di-oestrus was analysed (**c**). All bars represent mean ± standard deviation

In the placebo group, while di-oestrus was observed more frequently, the oestrus cycle was also observed (Supplementary Fig. 2), which does not explain female infertility in the human CVD model mice. The administration of probucol significantly increased the frequency of oestrus (37.3 ± 8.0%, mean ± SD, *P* < 0.05, Student’s *t*-test; Fig. 2a) compared to that in the placebo group (24.2 ± 7.3%). No differences were observed between the placebo and probucol groups in the histological analysis of the ovaries (Fig. 2b), including the follicular densities of each developmental stage (Fig. 2c). This suggests that female infertility in the human CVD model mouse was not caused by dysfunction in the fertility process during oocyte development.

### Assessment of Oocyte Maturation and Blastocyst Development

**Fig. 3:**
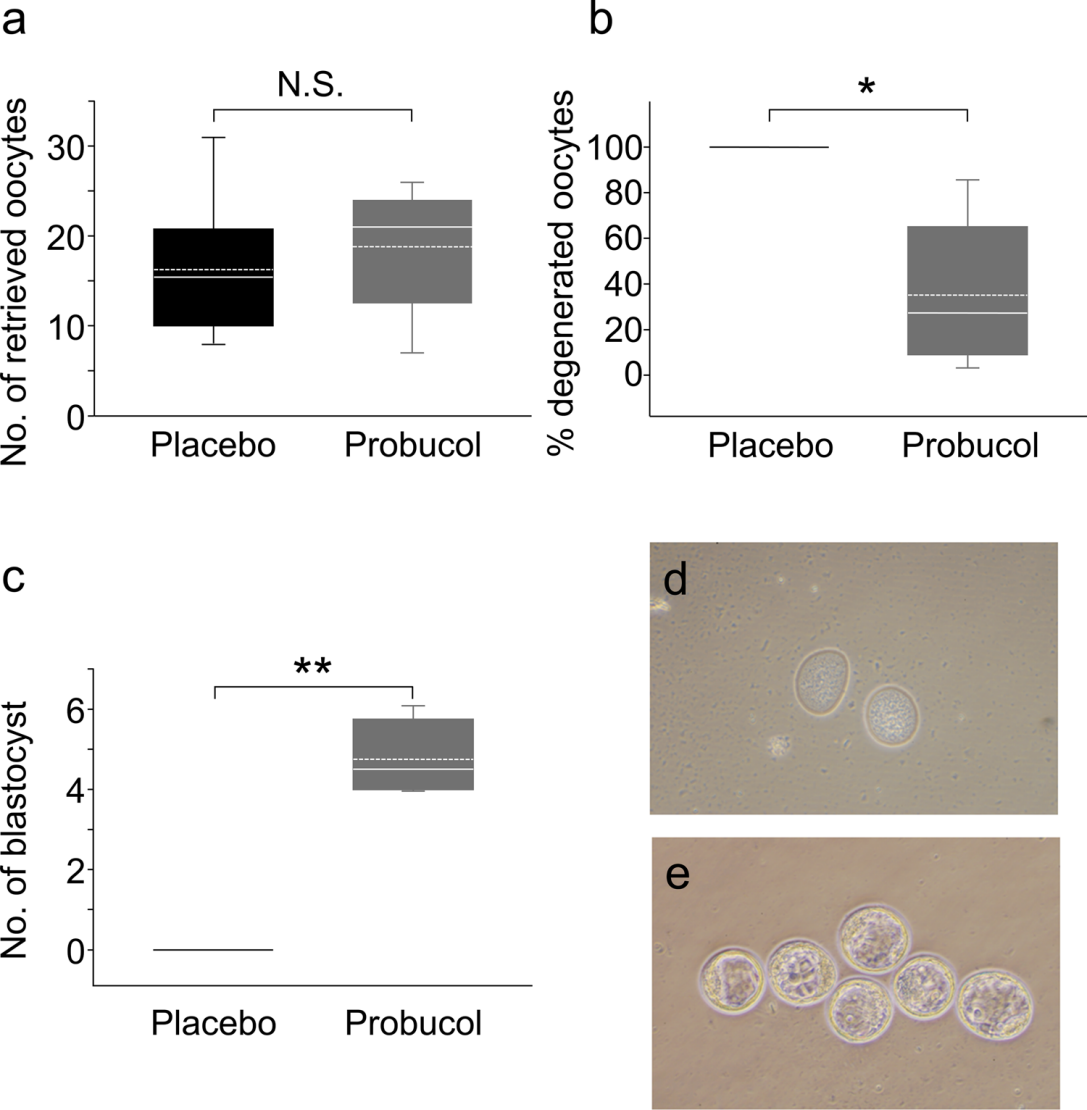
Analysis of oocyte maturation and blastocyst development. The box plots show the number of retrieved mature metaphase II oocytes after superovulation treatment (**a**), percentage of degenerated oocytes (**b**), and blastocysts that were flushed out from the uterus on day 4.0–4.5 post-coitus (**c**) in the SR-BI KO/*ApoeR61*^*h/h*^ mice with a normal chow diet (placebo group, black box) and a normal chow diet containing 0.5% probucol (probucol group, grey box). The horizontal line within the box shows the median, and the dotted line within the box shows the mean value. Data were evaluated using the Shapiro–Wilk normality test and Mann–Whitney U test (*P < 0.005, **P < 0.01)

During the ovulation period, oocytes resume meiosis [39]. Is there any dysfunctional fertility process during ovulation that is recovered by probucol administration in the human CVD model mouse? Overall, we found no significant difference in the number of retrieved oocytes after superovulation between the placebo (17.3 ± 7.7, mean ± SD) and probucol (18.8 ± 7.2) groups (Fig. 3a). However, most oocytes in the placebo group were degenerated (Fig. 3b). Administration of probucol significantly reduced the proportion of degenerated oocytes (*P* < 0.005, Mann–Whitney U test, Fig. 3b). On day 4.0–4.5 p.c., the blastocysts were flushed out of the uterus in the probucol group (4.8 ± 1.0, mean ± SD, *P* < 0.01, Mann–Whitney U test, Fig. 3c and e); in the placebo group, only degenerated oocytes were observed, with no blastocysts (Fig. 3c and d).

### Assessment of Uterine Receptivity

**Fig. 4:**
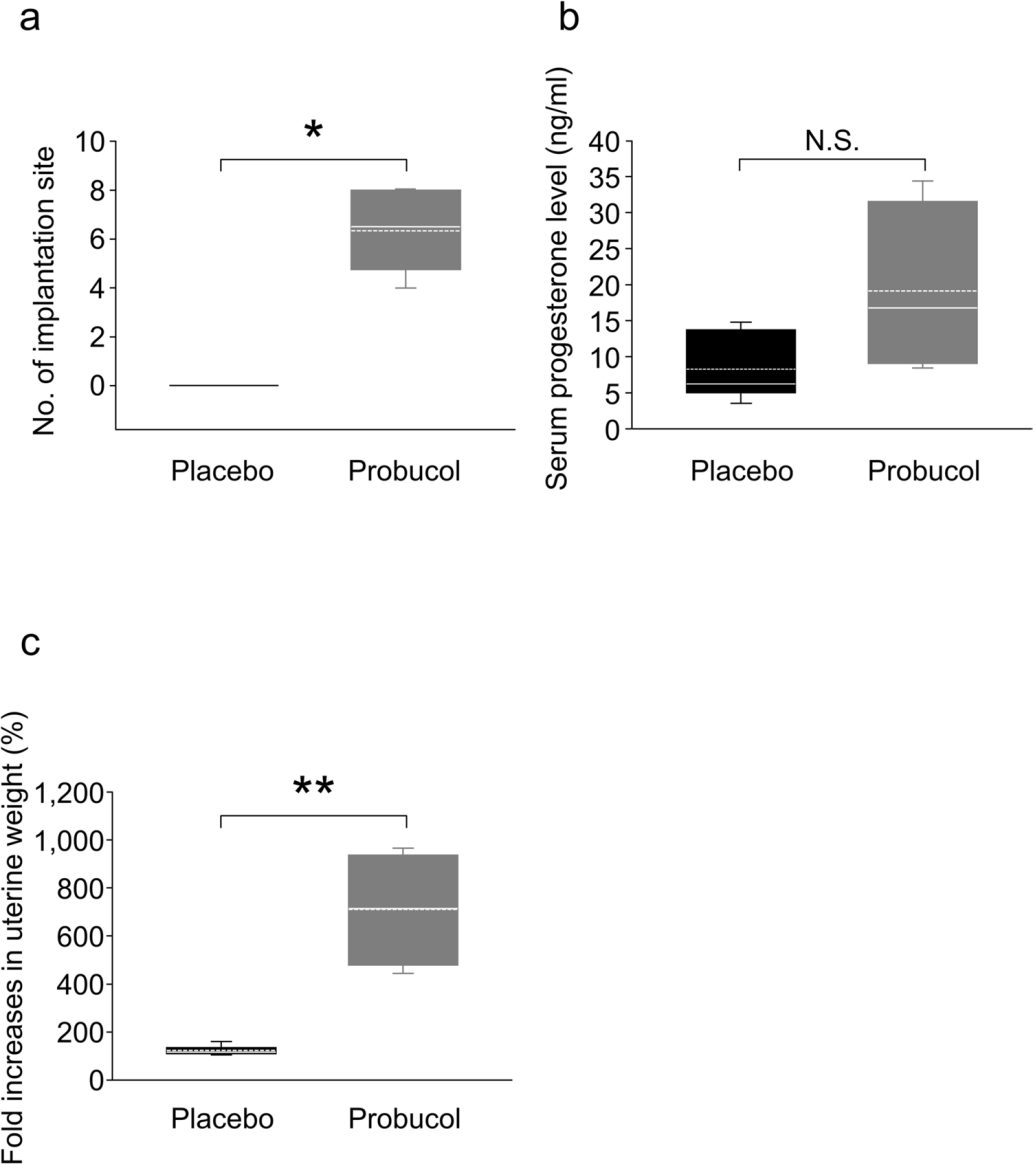
Serum progesterone level and decidualisation. The box plots show the number of implantation sites detected by an intravenous injection of Evans Blue on day 4.0–4.5 post-coitus (p.c.) (**a**), serum progesterone levels on day 4.5 p.c. (**b**) and the fold-increase of uterine weight at 72 h after sesame oil injection for mechanical decidualisation (**c**) in the SR-BI KO/*ApoeR61*^*h/h*^ mice with normal chow diet (placebo group; black box, *n* = 7) and normal chow diet containing 0.5% probucol (probucol group; grey box, *n* = 6). The horizontal line within the box shows the median, and the dotted line within the box shows the mean value. The data were evaluated using Mann–Whitney U test (*P < 0.001, **P < 0.05) or Student’s *t*-test with Shapiro–Wilk normality test

Implantation of the blastocyst into the maternal uterus is the first step of pregnancy. To ensure success of the implantation process, it is not only important that the appropriate conditions for the blastocysts are met, the appropriate uterine endometrium conditions are also essential. In the placebo group, no blastocysts developed and no implantation sites observed; however, this was partly rescued by the administration of probucol (Fig. 3b); furthermore, the administration of probucol significantly increased the number of implantation sites (6.3 ± 1.6, mean ± SD, *P* < 0.001, Mann–Whitney U test; Fig. 4a). Regarding the function on the uterine side, the serum progesterone levels tended to be increased in the probucol group (19.1 ± 6.0 ng/ml, mean ± SD) compared to those in the placebo group (8.3 ± 1.9 ng/ml). However, there was no significant difference because of the large variance in the data (*P* = 0.075, Student’s *t*-test; Fig. 4b). Decidualisation is one of the essential processes for implantation in the uterine endometrium. In the placebo group, following induction of artificial decidualisation, the uterine tissue weight was similar to that of the untreated uterine tissue (123.3 ± 8.0%, mean ± SD; Fig. 4c). Conversely, a decidual response was observed in the probucol group (710.0 ± 119.4%) and was significantly higher than that in the placebo group (*P* < 0.05, Mann–Whitney U test; Fig. 4c). This suggests that the impairment of the decidual response is at least one of the causes of the dysfunction of uterine receptivity in the human CVD model mouse.

### How Does the Dysfunction of Uterine Receptivity Occur?

**Fig. 5:**
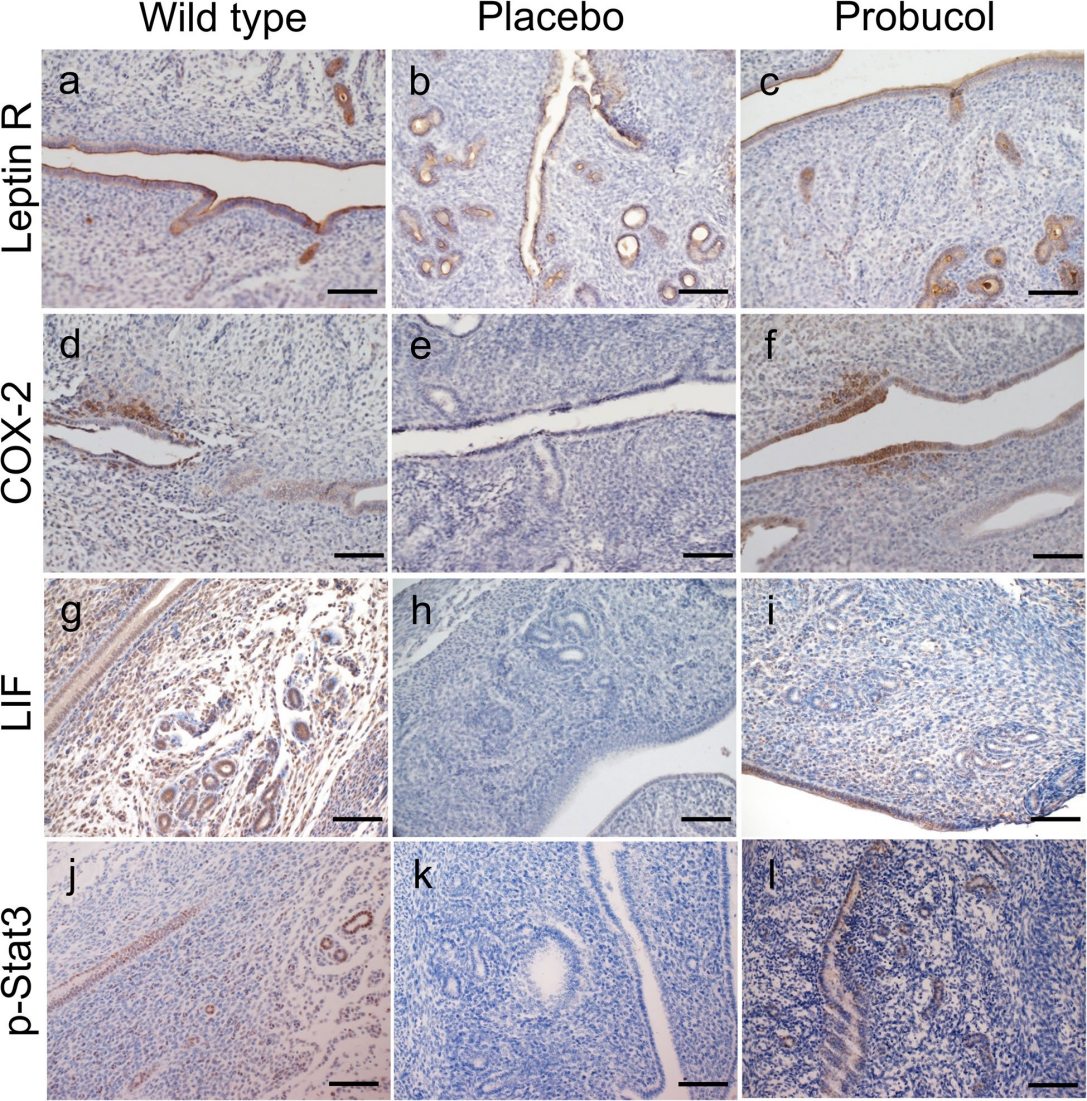
Immunohistochemical evaluation of uteri on day 4.5 post-coitus in SR-BI KO/*ApoeR61*^*h/h*^ mice. Leptin receptor (Leptin R) (**a-c**), cyclooxygenase (COX) −2 (**d-f**), leukaemia inhibitory factor (LIF) (**g-i**) and phospho-signal transducer and activator of transcription (Stat) −3 (**j-l**) immunostaining with haematoxylin counterstaining in SR-BI KO/*ApoeR61*^*h/h*^ mice with placebo (**b**, **e**, **h**, **k**) or probucol treatment (**c**, **f**, **i**, **l**). As a control, uteri from wild type mice (**a**, **d**, **g**, **j**) were evaluated. Scale bar = 100 µm

During the implantation period (day 4.5 post-coitus (p.c.; the morning on which vaginal plugging was first observed was designated as day 0.5 p.c.) in the wild-type and human CVD model mice, placebo, and probucol groups, the expression patterns of the leptin receptor in the uteri were similar in the three groups; the leptin receptor was expressed on both luminal and glandular epithelium (Fig. 5a, b, and c). The expression of COX-2 (Fig. 5d and f), LIF (Fig. 5g and i), and p-Stat3 (Fig. 5j and l) were all detected in both the luminal and glandular epithelium in the wild-type and probucol groups. However, this expression was not observed in the placebo group (Fig. 5e, h, and k).

## Discussion

The dysfunctional female fertility processes identified in the human CVD model mouse included degeneration of oocytes during the maturation processes prior to ovulation and impairment of implantation via dysfunctional decidualisation. This degeneration of oocytes during the maturation processes in the CVD model mice has also been reported in SR-BI-deficient mice [25, 40], which showed hypercholesterolemia under a low-fat diet but no spontaneous atherosclerosis, while apoE-deficient mice were fertile [41]. However, it is unclear whether this oocyte degeneration also occurs in women with infertility. Lipid metabolism has been considered important for oocyte maturation as the free fatty acids within the circulating blood enter the follicular fluid that surrounds the developing oocyte, and developing oocytes have endogenous stores of fatty acids in mammals [42]. However, oocyte lipid content varies in different species [42–44]. Multiple oocytes are retrieved at the same time during assisted reproductive technology (ART) treatment; however, it is extremely rare that the majority of the retrieved oocytes are degenerated.

COX-2, LIF, Stat3, and leptin receptor are known as molecules that play crucial roles in the uterine endometrium for implantation [45–49]. In the present study, decidualisation of the uterine endometrium was impaired in the human CVD model mice via down-regulations of COX-2, LIF, and p-Stat3 expression, but it was not related to the leptin receptor expression in the uterine endometrium. Progesterone is an indispensable signal for the uterus in the preparation for implantation in mammals, while the activation of Stat3 and the expression of leptin receptor, LIF, and COX-2 are affected by progesterone [45, 46]. In the present study, the progesterone level alone did not explain the dysfunctional uterine receptivity in the human CVD model mouse.

Dysregulated lipid metabolism is known to be one of the important contributing factors for CVD, while increased blood total cholesterol, HDL-C, and LDL-C are considered as indicators and even as treatment targets in clinical routine. Conversely, even though the associations between local and/or systematic lipid metabolism, oocyte maturation and uterine receptivity have been discussed for many years, they remain unelucidated [50]. After being fed an atherogenic diet rich in fat, cholesterol, and cholate, the human CVD model mice rapidly developed CVD and premature death (50% mortality: 33 ± 4.9 days) [29]. However, when fed a normal chow diet, they were healthy and active with body weights similar to that of wild-type mice, although they demonstrated significantly higher plasma levels of total cholesterol [29] as well as degeneration of oocytes during the maturation processes prior to ovulation and dysfunctional uterine receptivity. In the present study, the reductions of plasma total cholesterol, VLDL-C, and LDL-C levels and an increased plasma HDL-C level via administration of probucol ameliorated oocyte maturation and uterine receptivity. This suggests that systematic dysregulated lipid metabolism may affect uterine receptivity. This indicates that assessment of plasma total cholesterol levels and lipid profile may help propose a new infertility treatment in women with defective oocyte maturation and recurrent implantation failure and prevent future CVD in later-life.

Previous studies established that female infertility in SR-BI–deficient mice is associated with abnormal HDL particles, excess unesterified cholesterol accumulation in oocytes and eggs, and premature egg activation, and further demonstrated that probucol treatment can normalize lipid composition and restore fertility in SR-BI KO mice [28, 40, 51–54]. Our findings in SR-BI KO/ApoeR61h/h mice build upon this mechanistic framework and extend it to a disease-relevant cardiovascular model, highlighting that systemic lipid dysregulation may also impair later reproductive events, including blastocyst development, implantation, and decidualization.

## Conclusion

These results from the human CVD model mice show that female infertility via the degeneration of oocytes during the maturation processes prior to ovulation and the dysfunction of uterine receptivity may contribute to CVD in later-life. It is not possible to determine the degeneration of oocytes during the maturation process prior to ovulation without undergoing ART treatment and retrieving the eggs. Currently, dysfunction in uterine receptivity is considered as one of the important causes of female infertility, especially since the technology for preimplantation genetic testing for aneuploidy (PGT-A) has been applied for ART treatment worldwide. However, dysfunctional uterine receptivity is a hypothetical and pathophysiological condition with no definition or diagnosis method. Women with unexplained infertility with a history of recurrent implantation failure could be interesting candidates to assess future CVD risk. However, this population is challenging to identify using population registers in population-based cohort studies. A sizable long-term observational study in the population, sampled from infertility treatment data, could answer whether the dysfunction of uterine receptivity is a predisposition to CVD risk in later-life.

## Supplementary Information

Below is the link to the electronic supplementary material.

Supplementary file1 (TIF 2256 KB)

Supplementary file2 (TIF 2700 KB)

Supplementary file3 (DOCX 14 KB)

## References

[1] Green A, Beral V, Moser K. Mortality in women in relation to their childbearing history. BMJ. 1988;297(6645):391–5. 3408979 10.1136/bmj.297.6645.391 PMC1834270

[2] Ness RB, Harris T, Cobb J, Flegal KM, Kelsey JL, Balanger A, et al. Number of pregnancies and the subsequent risk of cardiovascular disease. N Engl J Med. 1993;328(21):1528–33. 10.1056/NEJM199305273282104. 8267704 10.1056/NEJM199305273282104

[3] Dekker JM, Schouten EG. Number of pregnancies and risk of cardiovascular disease. N Engl J Med. 1993;329(25):1893–4; author reply 4–5 8247048

[4] Lawlor DA, Emberson JR, Ebrahim S, Whincup PH, Wannamethee SG, Walker M, et al. Is the association between parity and coronary heart disease due to biological effects of pregnancy or adverse lifestyle risk factors associated with child-rearing? Findings from the British Women’s Heart and Health Study and the British Regional Heart Study. Circulation. 2003;107(9):1260–4. 12628945 10.1161/01.cir.0000053441.43495.1a

[5] Parikh NI, Cnattingius S, Dickman PW, Mittleman MA, Ludvigsson JF, Ingelsson E. Parity and risk of later-life maternal cardiovascular disease. Am Heart J. 2010;159(2):215–21. 10.1016/j.ahj.2009.11.017. 20152219 10.1016/j.ahj.2009.11.017

[6] Sanghavi M, Kulinski J, Ayers CR, Nelson D, Stewart R, Parikh N, et al. Association between number of live births and markers of subclinical atherosclerosis: the Dallas Heart Study. Eur J Prev Cardiol. 2016;23(4):391–9. 10.1177/2047487315571891. 25691547 10.1177/2047487315571891 PMC4827778

[7] Parikh NI, Cnattingius S, Mittleman MA, Ludvigsson JF, Ingelsson E. Subfertility and risk of later life maternal cardiovascular disease. Hum Reprod. 2012;27(2):568–75. 10.1093/humrep/der400. 22131387 10.1093/humrep/der400 PMC3283089

[8] Daan NM, Muka T, Koster MP, Roeters van Lennep JE, Lambalk CB, Laven JS, et al. Cardiovascular risk in women with premature ovarian insufficiency compared to premenopausal women at middle age. J Clin Endocrinol Metab. 2016;101(9):3306–15 10.1210/jc.2016-1141 10.1210/jc.2016-1141 27300572

[9] Gunning MN, Meun C, van Rijn BB, Daan NMP, van Roeters Lennep JE, Appelman Y, et al. The cardiovascular risk profile of middle age women previously diagnosed with premature ovarian insufficiency: a case-control study. PLoS One. 2020;15(3):e0229576. 10.1371/journal.pone.0229576. 32134933 10.1371/journal.pone.0229576 PMC7058320

[10] Gunning MN, Meun C, van Rijn BB, Maas A, Benschop L, Franx A, et al. Coronary artery calcification in middle-aged women with premature ovarian insufficiency. Clin Endocrinol (Oxf). 2019;91(2):314–22. 10.1111/cen.14003. 31049984 10.1111/cen.14003

[11] Bungum AB, Glazer CH, Arendt LH, Schmidt L, Pinborg A, Bonde JP, et al. Risk of hospitalization for early onset of cardiovascular disease among infertile women: a register-based cohort study. Hum Reprod. 2019;34(11):2274–81. 10.1093/humrep/dez154. 31665298 10.1093/humrep/dez154

[12] Wekker V, van Dammen L, Koning A, Heida KY, Painter RC, Limpens J, et al. Long-term cardiometabolic disease risk in women with PCOS: a systematic review and meta-analysis. Hum Reprod Update. 2020;26(6):942–60. 10.1093/humupd/dmaa029. 32995872 10.1093/humupd/dmaa029 PMC7600286

[13] Carson SA, Kallen AN. Diagnosis and management of infertility: a review. JAMA. 2021;326(1):65–76. 10.1001/jama.2021.4788. 34228062 10.1001/jama.2021.4788 PMC9302705

[14] Acton S, Rigotti A, Landschulz KT, Xu S, Hobbs HH, Krieger M. Identification of scavenger receptor SR-BI as a high density lipoprotein receptor. Science. 1996;271(5248):518–20. 8560269 10.1126/science.271.5248.518

[15] National Health Service (NHS) UK. Causes, infertility. Updated 2020/2/18. Available from: https://www.nhs.uk/conditions/infertility/causes/. Cited 2022 Dec 2022.

[16] Liao J, Huang W, Liu G. Animal models of coronary heart disease. J Biomed Res. 2017;31(1):3–10. 10.7555/JBR.30.20150051. 10.7555/JBR.30.20150051 PMC5274506 26585560

[17] Mahley RW. Apolipoprotein E: cholesterol transport protein with expanding role in cell biology. Science. 1988;240(4852):622–30. 3283935 10.1126/science.3283935

[18] Curtiss LK, Boisvert WA. Apolipoprotein E and atherosclerosis. Curr Opin Lipidol. 2000;11(3):243–51. 10882339 10.1097/00041433-200006000-00004

[19] Ishibashi S, Goldstein JL, Brown MS, Herz J, Burns DK. Massive xanthomatosis and atherosclerosis in cholesterol-fed low density lipoprotein receptor-negative mice. J Clin Invest. 1994;93(5):1885–93. 10.1172/JCI117179. 8182121 10.1172/JCI117179 PMC294295

[20] Plump AS, Smith JD, Hayek T, Aalto-Setala K, Walsh A, Verstuyft JG, et al. Severe hypercholesterolemia and atherosclerosis in apolipoprotein E-deficient mice created by homologous recombination in ES cells. Cell. 1992;71(2):343–53. 10.1016/0092-8674(92)90362-G. 1423598 10.1016/0092-8674(92)90362-g

[21] Zhang SH, Reddick RL, Piedrahita JA, Maeda N. Spontaneous hypercholesterolemia and arterial lesions in mice lacking apolipoprotein E. Science. 1992;258(5081):468–71. 10.1126/science.1411543. 1411543 10.1126/science.1411543

[22] Zhang SH, Reddick RL, Burkey B, Maeda N. Diet-induced atherosclerosis in mice heterozygous and homozygous for apolipoprotein E gene disruption. J Clin Invest. 1994;94(3):937–45. 10.1172/JCI117460. 8083379 10.1172/JCI117460 PMC295131

[23] Hoekstra M, Sorci-Thomas M. Rediscovering scavenger receptor type BI: surprising new roles for the HDL receptor. Curr Opin Lipidol. 2017;28(3):255–60. 10.1097/MOL.0000000000000413. 28301373 10.1097/MOL.0000000000000413 PMC5523812

[24] Rigotti A, Trigatti BL, Penman M, Rayburn H, Herz J, Krieger M. A targeted mutation in the murine gene encoding the high density lipoprotein (HDL) receptor scavenger receptor class B type I reveals its key role in HDL metabolism. Proc Natl Acad Sci USA. 1997;94(23):12610–5. 10.1073/pnas.94.23.12610. 9356497 10.1073/pnas.94.23.12610 PMC25055

[25] Trigatti B, Rayburn H, Vinals M, Braun A, Miettinen H, Penman M, et al. Influence of the high density lipoprotein receptor SR-BI on reproductive and cardiovascular pathophysiology. Proc Natl Acad Sci U S A. 1999;96(16):9322–7. 10430941 10.1073/pnas.96.16.9322 PMC17781

[26] Lund-Katz S, Phillips MC. High density lipoprotein structure-function and role in reverse cholesterol transport. Subcell Biochem. 2010;51:183–227. 10.1007/978-90-481-8622-8_7. 20213545 10.1007/978-90-481-8622-8_7 PMC3215094

[27] Braun A, Trigatti BL, Post MJ, Sato K, Simons M, Edelberg JM, et al. Loss of SR-BI expression leads to the early onset of occlusive atherosclerotic coronary artery disease, spontaneous myocardial infarctions, severe cardiac dysfunction, and premature death in apolipoprotein E-deficient mice. Circ Res. 2002;90(3):270–6. 11861414 10.1161/hh0302.104462

[28] Braun A, Zhang S, Miettinen HE, Ebrahim S, Holm TM, Vasile E, et al. Probucol prevents early coronary heart disease and death in the high-density lipoprotein receptor SR-BI/apolipoprotein E double knockout mouse. Proc Natl Acad Sci USA. 2003;100(12):7283–8. 10.1073/pnas.1237725100. 12771386 10.1073/pnas.1237725100 PMC165867

[29] Zhang S, Picard MH, Vasile E, Zhu Y, Raffai RL, Weisgraber KH, et al. Diet-induced occlusive coronary atherosclerosis, myocardial infarction, cardiac dysfunction, and premature death in scavenger receptor class B type I-deficient, hypomorphic apolipoprotein ER61 mice. Circulation. 2005;111(25):3457–64. 10.1161/CIRCULATIONAHA.104.523563. 15967843 10.1161/CIRCULATIONAHA.104.523563

[30] Nakaoka H, Nakagawa-Toyama Y, Nishida M, Okada T, Kawase R, Yamashita T, et al. Establishment of a novel murine model of ischemic cardiomyopathy with multiple diffuse coronary lesions. PLoS One. 2013;8(8):e70755. 10.1371/journal.pone.0070755. 23950999 10.1371/journal.pone.0070755 PMC3741297

[31] Pearson JT, Yoshimoto M, Chen YC, Sultani R, Edgley AJ, Nakaoka H, et al. Widespread coronary dysfunction in the absence of HDL receptor SR-B1 in an ischemic cardiomyopathy mouse model. Sci Rep. 2017;7(1):18108. 10.1038/s41598-017-18485-6. 29273789 10.1038/s41598-017-18485-6 PMC5741771

[32] Raffai RL, Weisgraber KH. Hypomorphic apolipoprotein E mice: a new model of conditional gene repair to examine apolipoprotein E-mediated metabolism. J Biol Chem. 2002;277(13):11064–8. 11792702 10.1074/jbc.M111222200

[33] Byers SL, Wiles MV, Dunn SL, Taft RA. Mouse estrous cycle identification tool and images. PLoS One. 2012;7(4):e35538. 10.1371/journal.pone.0035538. 22514749 10.1371/journal.pone.0035538 PMC3325956

[34] Usui S, Hara Y, Hosaki S, Okazaki M. A new on-line dual enzymatic method for simultaneous quantification of cholesterol and triglycerides in lipoproteins by HPLC. J Lipid Res. 2002;43(5):805–14. 11971952

[35] Tanaka A, Nakamura H, Tabata Y, Fujimori Y, Kumasawa K, Kimura T. Effect of sustained release of basic fibroblast growth factor using biodegradable gelatin hydrogels on frozen-thawed human ovarian tissue in a xenograft model. J Obstet Gynaecol Res. 2018;44(10):1947–55. 10.1111/jog.13726. 29998469 10.1111/jog.13726

[36] Gougeon A. Dynamics of follicular growth in the human: a model from preliminary results. Hum Reprod. 1986;1(2):81–7. 3558758 10.1093/oxfordjournals.humrep.a136365

[37] Huet YM, Dey SK. Role of early and late oestrogenic effects on implantation in the mouse. J Reprod Fertil. 1987;81(2):453–8. 3323496 10.1530/jrf.0.0810453

[38] Rider V, McRae A, Heap RB, Feinstein A. Passive immunization against progesterone inhibits endometrial sensitization in pseudopregnant mice and has antifertility effects in pregnant mice which are reversible by steroid treatment. J Endocrinol. 1985;104(1):153–8. 3968500 10.1677/joe.0.1040153

[39] Pritchett KR, Taft RA. Chapter 3 - Reproductive biology of the laboratory mouse. In: Fox JG, Davisson MT, Quimby FW, Barthold SW, Newcomer CE, Smith AL, editors. The mouse in biomedical research, volume III: normative biology, husbandry, and models. 2nd ed. Academic Press; 2007.

[40] Miettinen HE, Rayburn H, Krieger M. Abnormal lipoprotein metabolism and reversible female infertility in HDL receptor (SR-BI)-deficient mice. J Clin Invest. 2001;108(11):1717–22. 10.1172/JCI13288. 11733567 10.1172/JCI13288 PMC200987

[41] Zhang T, Dai P, Cheng D, Zhang L, Chen Z, Meng X, et al. Obesity occurring in apolipoprotein E-knockout mice has mild effects on fertility. Reproduction. 2014;147(2):141–51. 10.1530/REP-13-0470. 24196014 10.1530/REP-13-0470

[42] Bradley J, Swann K. Mitochondria and lipid metabolism in mammalian oocytes and early embryos. Int J Dev Biol. 2019;63:93–103. 10.1387/ijdb.180355ks. 31058306 10.1387/ijdb.180355ks

[43] Ferreira CR, Saraiva SA, Catharino RR, Garcia JS, Gozzo FC, Sanvido GB, et al. Single embryo and oocyte lipid fingerprinting by mass spectrometry. J Lipid Res. 2010;51(5):1218–27. 10.1194/jlr.D001768. 19965589 10.1194/jlr.D001768 PMC2853449

[44] McEvoy TG, Coull GD, Broadbent PJ, Hutchinson JS, Speake BK. Fatty acid composition of lipids in immature cattle, pig and sheep oocytes with intact zona pellucida. J Reprod Fertil. 2000;118(1):163–70. 10793638

[45] Cha J, Sun X, Dey SK. Mechanisms of implantation: strategies for successful pregnancy. Nat Med. 2012;18(12):1754–67. 10.1038/nm.3012. 23223073 10.1038/nm.3012 PMC6322836

[46] Dos Santos E, Serazin V, Morvan C, Torre A, Wainer R, de Mazancourt P, et al. Adiponectin and leptin systems in human endometrium during window of implantation. Fertil Steril. 2012;97(3):771–8 e1. 10.1016/j.fertnstert.2011.12.042 10.1016/j.fertnstert.2011.12.042 22265003

[47] Yoon SJ, Cha KY, Lee KA. Leptin receptors are down-regulated in uterine implantation sites compared to interimplantation sites. Mol Cell Endocrinol. 2005;232(1–2):27–35. 10.1016/j.mce.2005.01.002. 15737466 10.1016/j.mce.2005.01.002

[48] Nakamura H, Kimura T, Koyama S, Ogita K, Tsutsui T, Shimoya K, et al. Mouse model of human infertility: transient and local inhibition of endometrial STAT-3 activation results in implantation failure. FEBS Lett. 2006;580(11):2717–22. 10.1016/j.febslet.2006.04.029. 16647058 10.1016/j.febslet.2006.04.029

[49] Dimitriadis E, Sharkey AM, Tan YL, Salamonsen LA, Sherwin JR. Immunolocalisation of phosphorylated STAT3, interleukin 11 and leukaemia inhibitory factor in endometrium of women with unexplained infertility during the implantation window. Reprod Biol Endocrinol. 2007;5(1):44. 10.1186/1477-7827-5-44. 18047677 10.1186/1477-7827-5-44 PMC2217553

[50] von Grothusen C, Lalitkumar S, Boggavarapu NR, Gemzell-Danielsson K, Lalitkumar PG. Recent advances in understanding endometrial receptivity: molecular basis and clinical applications. Am J Reprod Immunol. 2014;72(2):148–57. 10.1111/aji.12226. 24635108 10.1111/aji.12226

[51] Yesilaltay A, Morales MG, Amigo L, Zanlungo S, Rigotti A, Karackattu SL, Donahee MH, Kozarsky KF, Krieger M. Effects of hepatic expression of the high-density lipoprotein receptor SR-BI on lipoprotein metabolism and female fertility. Endocrinology. 2006;147(4):1577–88. 16410302 10.1210/en.2005-1286

[52] Yesilaltay A, Dokshin GA, Busso D, Wang L, Galiani D, Chavarria T, Vasile E, Quilaqueo L, Orellana JA, Walzer D, Shalgi R, Dekel N, Albertini DF, Rigotti A, Page DC, Krieger M. Excess cholesterol induces mouse egg activation and may cause female infertility. Proc Natl Acad Sci USA. 2014;111(46):E4972–80. 25368174 10.1073/pnas.1418954111 PMC4246315

[53] Quiroz A, Molina P, Santander N, Gallardo D, Rigotti A, Busso D. Ovarian cholesterol efflux: ATP-binding cassette transporters and follicular fluid HDL regulate cholesterol content in mouse oocytes. Biol Reprod. 2020;102(2):348–61. 31423535 10.1093/biolre/ioz159

[54] Arias A, Quiroz A, Santander N, Morselli E, Busso D. Implications of high-density cholesterol metabolism for oocyte biology and female fertility. Front Cell Dev Biol. 2022;10:941539. 36187480 10.3389/fcell.2022.941539 PMC9518216

