# Supplementary figures and images for: Female Infertility and Risk for Later-Life Cardiovascular Disease: Lessons from a Mouse Model of Human Cardiovascular Disease
Source: Reprod Sci. 2026 Jan 16;33(2):457–66. doi: 10.1007/s43032-025-02026-y (PMC12992438; doi:10.1007/s43032-025-02026-y)

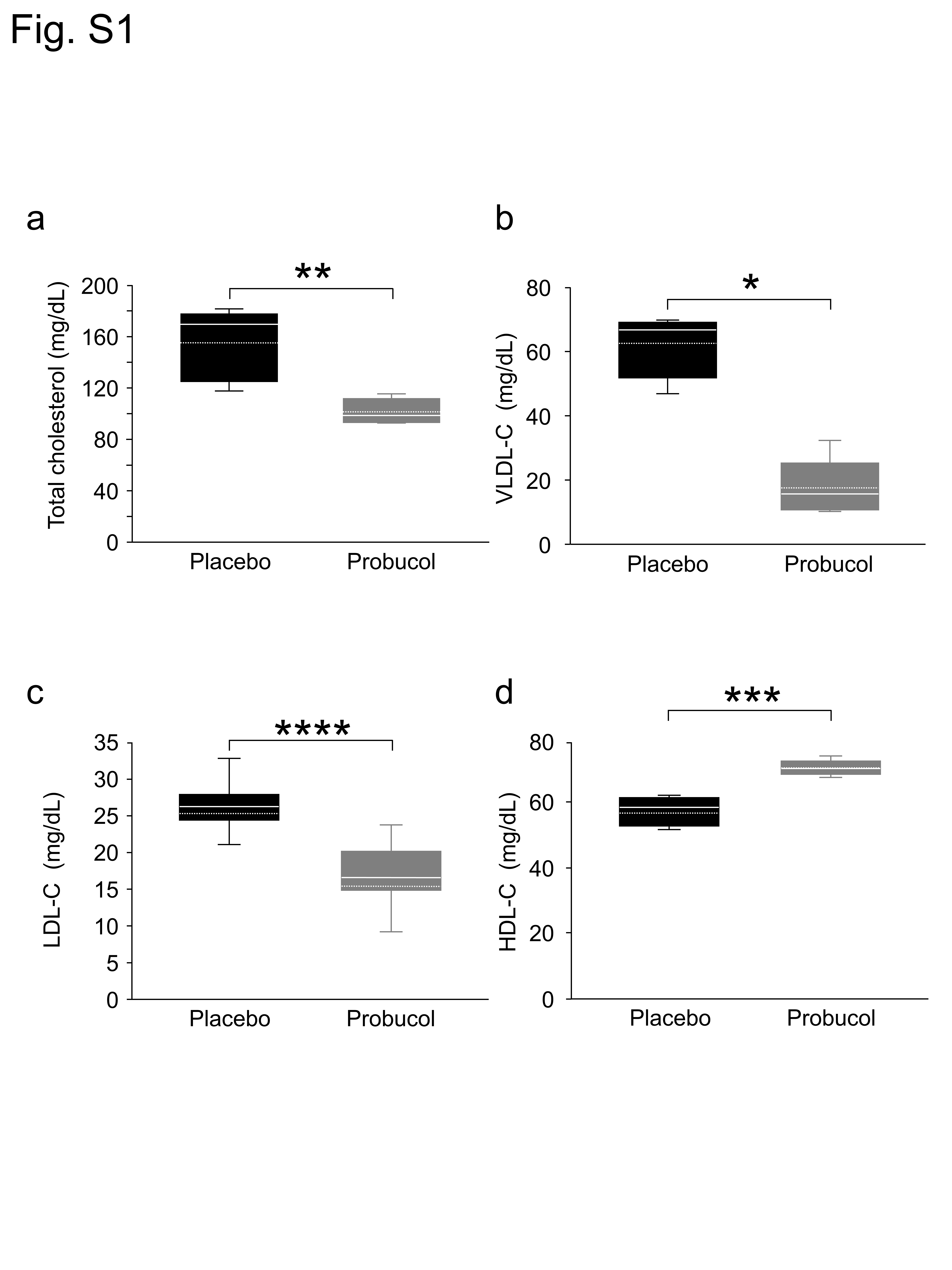

Supplement: Supplementary file 1 — Supplementary file1 (TIF 2256 KB) [file 43032_2025_2026_MOESM1_ESM.tif]

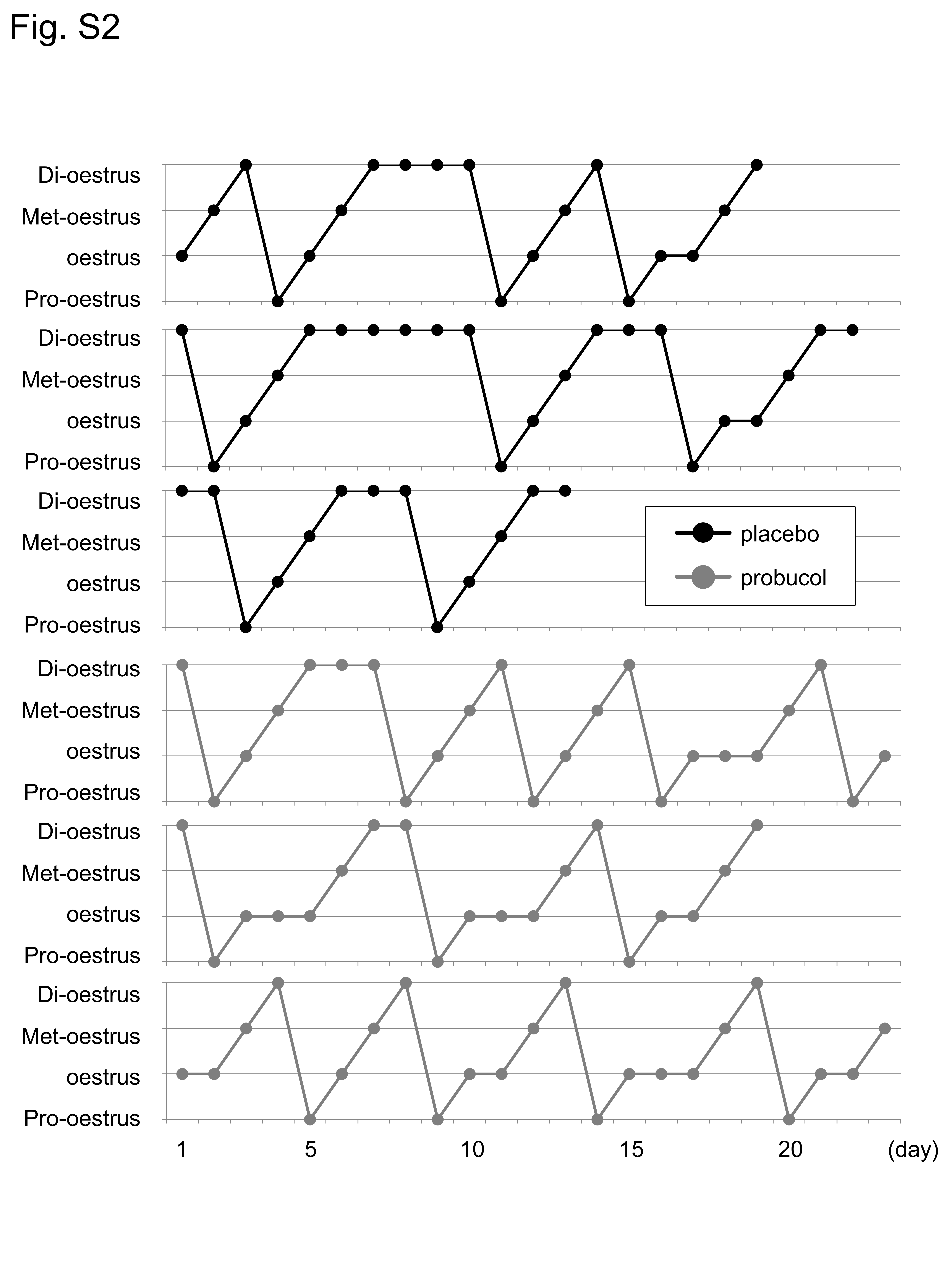

Supplement: Supplementary file 2 — Supplementary file2 (TIF 2700 KB) [file 43032_2025_2026_MOESM2_ESM.tif]
