# Supplementary material for: Female Infertility and Risk for Later-Life Cardiovascular Disease: Lessons from a Mouse Model of Human Cardiovascular Disease
Source: Reprod Sci. 2026 Jan 16;33(2):457–66. doi: 10.1007/s43032-025-02026-y (PMC12992438; doi:10.1007/s43032-025-02026-y)
Supplement: Supplementary file 3 — Supplementary file3 (DOCX 14 KB) [file 43032_2025_2026_MOESM3_ESM.docx]

**Supplementary materials**

**Supplementary Fig. S1.**

Plasma lipoprotein total cholesterol profiles.

Box plot showing plasma total cholesterol level (A), VLDL-C (B), LDL-C (D) and HDL-C (E) in the SR-BI KO/ApoeR61^h/h^ mice with a normal chow diet (placebo group; black box) and a normal chow diet containing 0.5% probucol (probucol group; grey box). The horizontal line within the box shows the median and the dotted line shows the mean value. Data were evaluated using Shapiro–Wilk normality test and Student’s *t*-test (**P* < 0.001, ***P* < 0.005, ****P* < 0.01, *****P* < 0.05).

Abbreviations: VLDL-C, very low-density lipoprotein-cholesterol; LDL-C, low-density lipoprotein-cholesterol; HDL-C, high-density lipoprotein-cholesterol

**Supplementary Fig. S2.**

Sexual phase determined using vaginal smears on consecutive days.
